# Sorting nexin-4 regulates β-amyloid production by modulating β-site-activating cleavage enzyme-1

**DOI:** 10.1186/s13195-016-0232-8

**Published:** 2017-01-21

**Authors:** Na-Young Kim, Mi-Hyang Cho, Se-Hoon Won, Hoe-Jin Kang, Seung-Yong Yoon, Dong-Hou Kim

**Affiliations:** 1 Alzheimer’s Disease Experts Lab (ADEL), Asan Medical Center, University of Ulsan College of Medicine, Seoul, Korea; 2 Department of Brain Science, University of Ulsan College of Medicine, 88, Olympic-ro 43-gil, SongPa-Gu, Seoul 05505 Korea; 3 Bio-Medical Institute of Technology (BMIT), University of Ulsan College of Medicine, Seoul, Korea; 4 Cell Dysfunction Research Center (CDRC), University of Ulsan College of Medicine, Seoul, Korea

**Keywords:** Alzheimer’s disease, Sorting nexin, BACE1, APP, Lysosome

## Abstract

**Background:**

Amyloid precursor protein (APP) is cleaved by β-site amyloid precursor protein-cleaving enzyme 1 (BACE1) to produce β-amyloid (Aβ), a critical pathogenic peptide in Alzheimer’s disease (AD). Aβ generation can be affected by the intracellular trafficking of APP or its related secretases, which is thus important to understanding its pathological alterations. Although sorting nexin (SNX) family proteins regulate this trafficking, the relevance and role of sorting nexin-4 (SNX4) regarding AD has not been studied yet.

**Methods:**

In this study, human brain tissue and APP/PS1 mouse brain tissue were used to check the disease relevance of SNX4. To investigate the role of SNX4 in AD pathogenesis, several experiments were done, such as coimmunoprecipitation, Western blotting, immunohistochemistry, and gradient fractionation.

**Results:**

We found that SNX4 protein levels changed in the brains of patients with AD and of AD model mice. Overexpression of SNX4 significantly increased the levels of BACE1 and Aβ. Downregulation of SNX4 had the opposite effect. SNX4 interacts with BACE1 and prevents BACE1 trafficking to the lysosomal degradation system, resulting in an increased half-life of BACE1 and increased production of Aβ.

**Conclusions:**

We show that SNX4 regulates BACE1 trafficking. Our findings suggest novel therapeutic implications of modulating SNX4 to regulate BACE1-mediated β-processing of APP and subsequent Aβ generation.

**Electronic supplementary material:**

The online version of this article (doi:10.1186/s13195-016-0232-8) contains supplementary material, which is available to authorized users.

## Background

Alzheimer’s disease (AD) is a progressive neurodegenerative disorder characterized by senile plaques containing extracellular deposits of the β-amyloid (Aβ) peptide [1]. The Aβ_40–42_ peptide is derived from the amyloid precursor protein (APP) via the action of two membrane-bound proteolytic enzymes: β- and γ-secretase. β-site amyloid precursor protein-cleaving enzyme 1 (BACE1) is a transmembrane aspartyl protease that mediates the β-secretase cleavage, yielding a soluble ectodomain-secreted APP derivative (sAPPβ), as well as to a membrane-anchored C-terminal fragment (CTF) that subsequently undergoes presenilin-mediated γ-secretase cleavage [2–4]. The γ-secretase cleavage of CTF generates Aβ [5]. Previous reports have shown that APP proteolytic processing occurs at various subcellular sites. Aβ is produced in the *trans*-Golgi network (TGN), Golgi-associated vesicles, the endosomal system, and the endoplasmic reticulum/intermediate compartment [6–9].

BACE1 has been shown to transit through the secretory pathway and target the endosomal system, cycling between endosomes and the cell surface, probably via TGN [10, 11]. The critical and initial point of Aβ generation is mediated by BACE1; hence, much effort has been made to develop BACE1 inhibitors. It is thus important to understand which molecular machinery regulates the trafficking of BACE1 affecting Aβ generation.

Sorting nexins (SNXs) are a diverse group of cellular trafficking proteins that contain a phospholipid-binding motif (PX). SNXs can form protein-protein complexes and bind specific phospholipids, which suggests a role for these proteins in membrane trafficking and protein sorting [12–14]. The mammalian SNX protein containing a Bin/amphiphysin/Rvs domain (SNX-BAR) retromer is composed of two subcomplexes; a membrane remodeling unit comprising a specific combination of the SNX-BAR domains, including dimers of SNX1/SNX2 and SNX5/SNX6; and a stable trimeric complex of vacuolar protein sorting (VPS) proteins. The trimer of VPS26-VPS29-VSP35 provides cargo selectivity through direct binding of VPS35 to the cytosolic tail of several cargo proteins (e.g., cation-independent mannose-6-phosphate receptor) [15]. The assembly of these two subcomplexes allows the SNX-BAR retromer to coordinate the formation/stabilization of endosomal tubules selectively enriched with the appropriate cargo for endosome-to-TGN retrieval [16, 17].

Recent studies involving sorting nexin-4 (SNX4) regulation of the transferrin receptor have suggested that SNX-BARs play a fundamental, evolutionarily conserved role in tubule-based endosomal sorting [18–20]. Several SNX family members have been found to modulate Aβ generation through different regulatory mechanisms [21–23]. However, the functional roles of over 30 other mammalian SNX proteins remain unknown and deserve further investigation, particularly regarding their potential involvement in AD. In our present study, we first demonstrate that one of the SNX family members, SNX4, can interact with BACE1 and affect its intracellular trafficking, thereby mediating the β-processing of APP in Aβ production.

## Methods

### Human brain tissue and APP/PS1-transgenic mouse brain tissue

Mediotemporal gyri from eight patients with AD and seven age- and sex-matched controls were provided by the Netherlands Brain Bank (Table 1). Pathological staging of AD was based on the Braak staging system [24]. Hippocampi and cortices from age-matched (6 and 24 months) control and APP/PS1 mice were analyzed by Western blotting.

**Table 1:** Human mediotemporal gyrus samples used in this study

Diagnosis	Sex	Age (years)	Braak stage	Amyloid	PMD (h:minutes)	pH	Weight (g)
Alzheimer’s disease	M	85	5	C	07:10	6.13	1020
Alzheimer’s disease	M	65	6	C	08:50	6.88	1057
Alzheimer’s disease	M	65	5	C	05:50	6.36	1355
Alzheimer’s disease	M	65	5	C	07:20	6.47	1173
Alzheimer’s disease	M	87	5	C	06:10	6.14	1047
Alzheimer’s disease	M	67	5	C	04:10	6.40	1252
Alzheimer’s disease	M	70	6	C	04:50	6.95	1040
Alzheimer’s disease	M	82	5	C	05:15	6.34	1182
Nondemented control	M	73	0	O	24:45	?	1267
Nondemented control	M	71	1	O	07:40	6.20	1150
Nondemented control	M	87	1	A	10:20	6.32	1256
Nondemented control	M	80	0	O	07:15	5.80	1331
Nondemented control	M	84	1	A	05:35	6.98	1337
Nondemented control	M	82	1	O	05:10	6.75	1087
Nondemented control	M	78	1	O	<17:40	6.52	1125

Mediotemporal gyri from eight patients with AD and seven age- and sex-matched controls were provided by the Netherlands Brain Bank. Braak stages based on neurofibrillary tangles were 5 or 6 in subjects with Alzheimer’s disease (AD) and 0 or 1 in the controls [24]. Braak stages based on amyloid plaques were C in AD cases and 0 or A in the controls [24]. Tissue preparation time from death is displayed as postmortem delay (PMD)

### Plasmids

Human *SNX4* (GenBank accession number NM_003794) was tagged with green fluorescent protein (GFP) at its N-terminus for fluorescence imaging. These modified *SNX4* complementary DNAs were subcloned into a mammalian expression vector, *pEGFP-C1* (Invitrogen, Carlsbad, CA, USA). The sequence of all constructs was verified by DNA sequencing. All experiments were performed in SH-SY5Y, HeLa, and HEK293 cells or mouse primary cortical neurons.

### Cell culture and isolation of primary mouse cortical neurons

SH-SY5Y, HeLa, and HEK293 cells were maintained in DMEM (Thermo Fisher Scientific, Rockford, IL, USA) supplemented with 10% FBS (Thermo Fisher Scientific, Rockford, IL, USA) and incubated in 5% CO_2_ at 37 °C. Cultures of primary cortical neurons were prepared from the brains of embryonic day 16 pups as described previously [25]. Briefly, cerebral cortices were dissected in cold calcium- and magnesium-free Hanks’ balanced salt solution and incubated with a 0.125% trypsin solution for 15 minutes at 37 °C. Trypsin was inactivated with DMEM containing 20% FBS, and cortical tissue was dissociated by repeated trituration using a Pasteur pipette. Cell suspensions were diluted in neurobasal medium supplemented with Gibco B-27 components (Life Technologies/Thermo Fisher Scientific, Grand Island, NY, USA) and seeded onto plates coated with poly-d-lysine (catalogue number P7886-100MG; Sigma-Aldrich, St. Louis, MO, USA) and laminin (1 mg/ml; Life Technologies/Thermo Fisher Scientific, Grand Island, NY, USA). Neurons were maintained at 37 °C in a humidified 5% CO_2_ environment. All animal protocols used in this study were approved by Asan Institute for Life Sciences Animal Care and Use Committee.

### Transfection of plasmids and small interfering RNA

The SH-SY5Y, HeLa, and HEK293 cells and primary mouse cortical neurons were transfected with plasmids, scrambled small interfering RNA (siCTL), or a small interfering RNA (siRNA) mixture (siSNX4) of three different siRNAs designed for targeting to SNX4 using Lipofectamine 2000 reagent (catalogue number 11668-019; Invitrogen, Carlsbad, CA, USA) according to the manufacturer’s guide.

The following are sequences of the siRNAs targeting human SNX4:

1. Sense: 5′-CAGAUCAGUUAAAGAGUA-3′, antisense: 5′-UACUCUUUUAACUGAUCUG-3′
2. Sense: 5′-CAGAAUAAAGGUGCUAGAA-3′, antisense: 5′-UUCUAGCACCUUUAUUCUG-3′
3. Sense: 5′-GUUUCAAGACCAGCUGUUU-3′, antisense: 5′AAACAGCUGGUCUUGAAAC-3′

The following are sequences of the siRNAs targeting murine SNX4:

1. Sense: 5′-UGAAUGGAGUGCCAUCGAA-3′, antisense: 5′-UUCGAUGGCACUCCAUUCA-3′
2. Sense: 5′-GGAAUUCAGGUUUGGACCA-3′, antisense: 5′-UGGUCCAAACCUGAAUUCC-3′
3. Sense: 5′-GAGUAGCAGAUCGACUCUA-3′, antisense: 5′-UAGAGUCGAUCUGCUACUC-3′

### Immunocytochemistry and immunohistochemistry

For immunocytochemistry, SH-SY5Y and HeLa cells were plated onto 18-mm coverslips (Marienfeld, Lauda-Königshofen, Germany) coated with 0.05 mg/ml poly-d-lysine (Sigma-Aldrich, St. Louis, MO, USA). HeLa cells were transfected with *pEGFP-C1-SNX4*. At 24 h after transfection, cells were fixed with 4% paraformaldehyde. After being washed three times with PBS, cells were permeabilized with PBS containing 0.1% Triton X-100 for 5 minutes at room temperature (RT). Next, the cells were washed three times and blocked with PBS containing 5% bovine serum albumin for 30 minutes at 37 °C. The cells were washed three additional times and incubated with a primary antibody against rat hemagglutinin (HA) (Roche, Basel, Switzerland) for 60 minutes at 37 °C. After cells were washed five times with PBS, a secondary antibody coupled to Texas Red (Invitrogen, Carlsbad, CA, USA) was added for 60 minutes at 37 °C. Finally, the cells were washed five times and mounted for imaging. The cells were examined by confocal microscopy with the LSM 780 microscope (Carl Zeiss, Oberkochen, Germany), and image processing was performed using the ZEN software system (Carl Zeiss, Oberkochen, Germany).

For immunohistochemistry, paraffin-embedded blocks were sectioned and attached to the slide glasses. The paraffin of sectioned tissues was removed with xylene deparaffinizing solution. Next, tissues were dehydrated with various ethanol solutions at 100%, 90%, 80%, 70%, and 50% and washed twice with distilled water. For antigen retrieval, tissues were boiled for 5 minutes in 1 mM ethylenediaminetetraacetic acid (EDTA) solution (pH 8.0). After being washed three times with PBS, tissues were permeabilized with PBS containing 0.1% Triton X-100 for 20 minutes at RT. The cells were washed three times and were blocked with PBS containing 2% bovine serum albumin and 2% horse serum for 30 minutes at 37 °C. Tissues were washed three times and were incubated with a primary antibody against goat anti-SNX4 (Santa Cruz Biotechnology, Dallas, TX, USA), rabbit anti-BACE1 (Cell Signaling Technology, Danvers, MA, USA), mouse anti-BACE1 (Santa Cruz Biotechnology, Dallas, TX, USA), and rabbit anti-Rab11 (Cell Signaling Technology, Danvers, MA, USA). After tissues were washed five times with PBS, a secondary antibody coupled to fluorescein isothiocyanate and Texas Red (Invitrogen, Carlsbad, CA, USA) was added for 60 minutes at 37 °C. After that step, tissues were washed three times with PBS and mounted for imaging. The cells and tissue were examined by confocal microscopy with the LSM 780 microscope, and image processing was performed using the ZEN software system.

### Cell surface biotinylation assay

Cells transfected with *SNX4* were cooled on ice and washed three times with ice-cold PBS containing 1 mM MgCl_2_ and 0.1 mM CaCl_2_ to remove any contaminating proteins. After washing cells twice more with PBS, 0.5 mg of EZ-Link Sulfo-NHS-LC-Biotin (Thermo Fisher Scientific, Rockford, IL, USA) per milliliter of reaction volume was added and incubated at 4 °C for 60 minutes. After further washing cells twice with PBS, the cells were harvested in PBS and lysed in lysis buffer (1% Nonidet P-40, 40 mM Tris-HCl, pH 7.5, 150 mM NaCl, 10 mM EDTA, 5 mM ethylene glycol-bis(β-aminoethyl ether)-*N,N,N′,N′*-tetraacetic acid, 5% glycerol, 1 mM phenylmethylsulfonyl fluoride, and protease inhibitor cocktail [EMD Millipore, Billerica, MA, USA]). Cell lysates were centrifuged at 14,499 × *g* for 10 minutes at 4 °C to remove any insoluble material. The resulting supernatant was incubated with 50 μl of 50% streptavidin-coated agarose beads (Thermo Fisher Scientific, Rockford, IL, USA) with rotation for 2 h at 4 °C. After the beads were washed three times with lysis buffer, the bound proteins were eluted with SDS sample buffer by boiling for 5 minutes. Total protein and isolated biotinylated proteins were analyzed by immunoblotting. Glyceraldehyde 3-phosphate dehydrogenase (GAPDH) in the surface fraction was used as a negative control to confirm fractionation [26, 27].

### Coimmunoprecipitation and Western blot analysis

For coimmunoprecipitation and immunoblotting, HEK293 cells or cultured mouse cortical neurons transiently expressing *BACE1-HA* and *GFP* (mock) or *GFP-SNX4* construct or mouse brain tissues were lysed with lysis buffer for 1 h at 4 °C. Cell lysates were centrifuged at 14,499 × *g* for 10 minutes at 4 °C to remove any insoluble material. Immunoprecipitation was performed by overnight incubation with anti-BACE1 antibody (Cell Signaling Technology, Danvers, MA, USA), anti-GFP (Roche, Basel, Switzerland), or anti-HA (Roche, Basel, Switzerland) antibody. Immune complexes were captured using protein G sepharose (GE Healthcare Life Sciences, Piscataway, NJ, USA), followed by washing with lysis buffer three times. Immunoprecipitated samples or 5% of the input lysates were used for immunoblotting. For Western blot analysis, protein lysates from HEK293 cells or primary mouse cortical neurons or mouse brain tissue were homogenized in 1× IGEPAL (I8896; Sigma-Aldrich, St. Louis, MO, USA), a protein extraction solution, according to the manufacturer’s instructions and incubated at −20 °C with rotation for 30 minutes. The suspension was microcentrifuged at 15,682 × *g* for 15 minutes at 4 °C, and the supernatant was collected. Protein concentrations were measured by Bradford assay, and proteins were mixed with 5× sample buffer (60 mM Tris-HCl, pH 6.8, 2% wt/vol SDS, 25% vol/vol glycerol, 14.4 mM vol/vol β-mercaptoethanol, and bromophenol blue), boiled at 100 °C for 5 minutes, and stored at −20 °C. Proteins were resolved by SDS-PAGE at a constant voltage (110 V) and transferred at 100 V for 1.5 h to polyvinylidene difluoride membranes (0.2-mm pore size; Bio-Rad Laboratories, Hercules, CA, USA). After 1-h incubation in blocking buffer (PBS containing 0.1% vol/vol Tween-20 [PBST]) containing 3% wt/vol bovine serum albumin and 5% vol/vol skim milk, blots were incubated with primary antibodies overnight at 4 °C. Blots were next washed in PBST buffer, incubated with HRP-conjugated anti-immunoglobulin G (1:5000; Thermo Fisher Scientific, Rockford, IL, USA), and visualized using enhanced chemiluminescence reagents (GE Healthcare Bio-Sciences, Pittsburgh, PA, USA) and x-ray film. The primary antibodies and dilutions used in the Western blot analysis were goat anti-SNX4 (1:500; Santa Cruz Biotechnology, Dallas, TX, USA), rabbit anti-BACE1 (1:5000; Abcam, Cambridge, UK), mouse anti-GFP (1:5000; Santa Cruz Biotechnology, Dallas, TX, USA), mouse anti-β-actin (1:10,000; Sigma-Aldrich, St. Louis, MO, USA), mouse anti-GAPDH (1:2000; EMD Millipore, Billerica, MA, USA), mouse anti-early endosome antigen 1 (EEA1) (1:2000; BD Biosciences, San Jose, CA, USA), rabbit anti-Rab7 (1:2000; Sigma-Aldrich, St. Louis, MO, USA), rabbit anti-Rab11 (1:1000; Cell Signaling Technology, Danvers, MA, USA), and mouse anti-Aβ (6E10, 1:5000; Covance, Princeton, NJ, USA). The band intensities were measured and analyzed with ImageJ software (National Institutes of Health, Bethesda, MD, USA).

### Protein synthesis inhibition

HEK293 cells stably expressing BACE1 were transfected with mock or *SNX4* constructs for 24 h, followed by treatment with 10 μg/ml cycloheximide, a protein synthesis inhibitor (Sigma-Aldrich, St. Louis, MO, USA) for 0, 1, and 6 h.

### Gradient fractionation

To produce a single-cell suspension, SH-SY5Y cells were plated in 10 ml of DMEM (Thermo Fisher Scientific, Rockford, IL, USA) supplemented with 5% FBS (Thermo Fisher Scientific, Rockford, IL, USA) and grown to 90% confluency with 5% CO_2_ at 37 °C. SH-SY5Y cells were cotransfected with *BACE1* and *SNX4* constructs or mock treatment for 48 h. After transfection, the cells were washed three times with cold PBS and harvested. The suspension was microcentrifuged at 13,362 × *g* for 5 minutes at 4 °C, and the supernatant was removed. In accordance with the manufacturer’s instructions, the pellet was resuspended in gradient fractionation solution A (catalogue number 89839; Thermo Fisher Scientific, Rockford, IL, USA) and incubated with added protease inhibitor at −4 °C for 2 minutes. The mixed solution was homogenized on ice, added to gradient fractionation solution B, and centrifuged at 500 × *g* for 10 minutes at 4 °C. The supernatant was transferred to a 1.5-ml tube, and protein concentrations were measured by the Bradford method. Equal amounts of protein were mixed with OptiPrep gradient solution (Sigma-Aldrich, St. Louis, MO, USA). The supernatant containing total protein in 640 μl was loaded onto the top of a step gradient composed of 320 μl of 30% gradient, 320 μl of 27% gradient, 160 μl of 23% gradient, 320 μl of 20% gradient, and 160 μl of 17% gradient (total approximately 2 ml). Gradients were centrifuged at 145,000 × *g* for 2 h at 4 °C. Fourteen 130-μl fractions were collected from the top of the gradient. The distributions of BACE1, EEA1, Rab7, Rab11, and β-actin along the gradient were assayed by SDS-PAGE followed by Western blot analysis.

### Quantitative analysis of fluorescence intensity

The fluorescence intensity of immunostained cells in images were measured using the ZEN 2011 software system (blue edition). Each intensity value was corrected with background fluorescence intensity of nonstained cells and normalized by each control. The intensity value in all figures was analyzed from about 50–100 cells, and then *p* values were calculated using Student’s *t* test.

### Statistical analysis

Data are presented as mean ± SEM and were analyzed using Student’s *t* test. A *p* value less than 0.05 was considered to be statistically significant.

## Results

### Altered levels of SNX4 in the brains of patients with AD and APP/PS1 mice

**Fig. 1:**
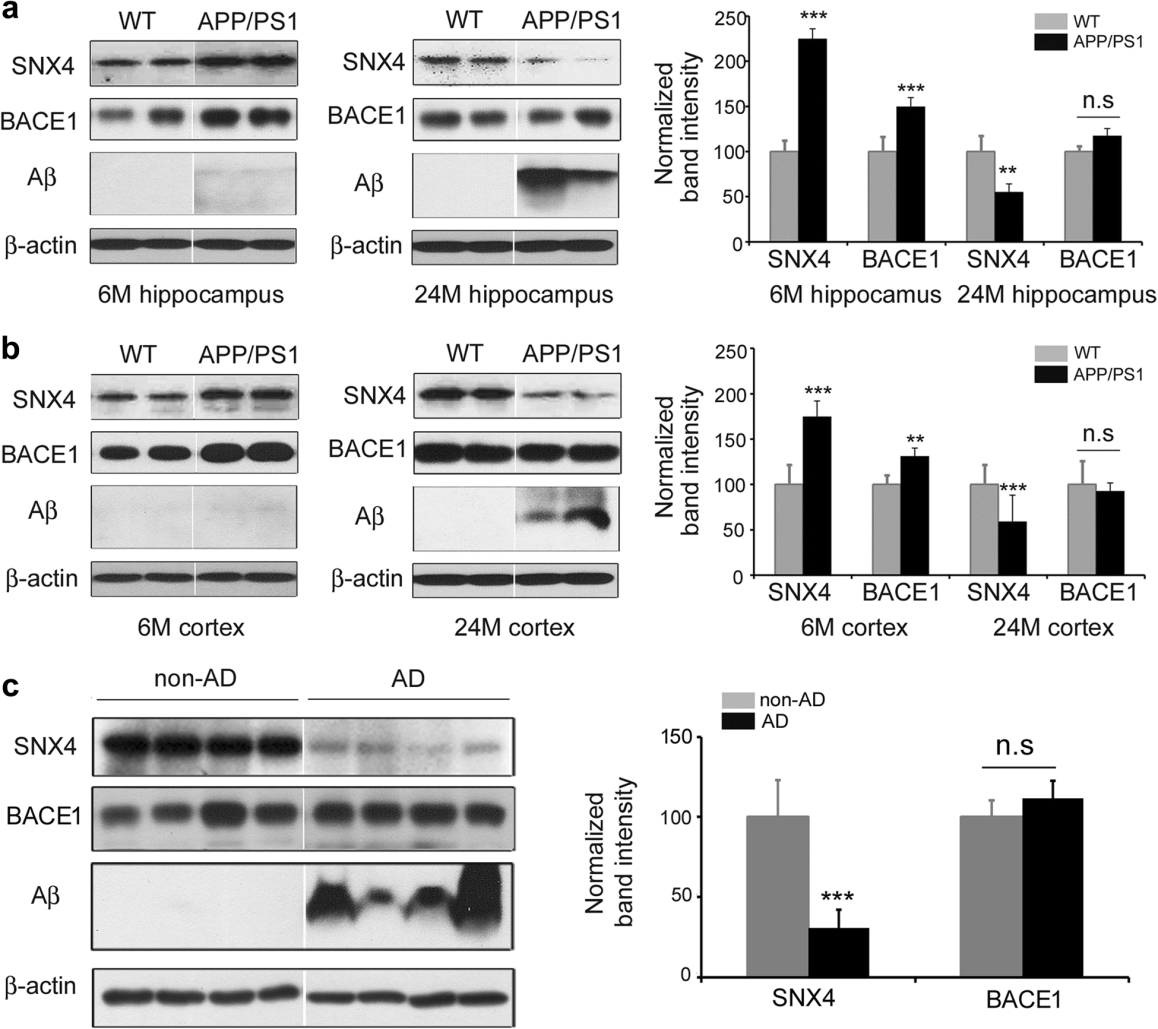
Altered levels of sorting nexin-4 (SNX4) in the brains of patients with Alzheimer’s disease (AD) and APP/PS1 mice. **a** Expression of SNX4 in the hippocampus of wild-type (WT) (*n* = 5) and APP/PS1 (*n* = 5) mice aged 6 months (6 M) and 24 months (24 M) analyzed by Western blotting with antibodies against SNX4, β-site amyloid precursor protein-cleaving enzyme 1 (BACE1), β-amyloid (Aβ), and β-actin. Representative images and graphs are shown. Data are presented as mean ± SEM. **p < 0.01, ***p < 0.001. *n.s* Not significant **b** Expression of SNX4 in the cortex of WT (*n* = 5) and APP/PS1 (*n* = 5) mice aged 6 months and 24 months analyzed by Western blotting with antibodies against SNX4, BACE1, Aβ, and β-actin. Representative images and graphs are shown. Data are presented as mean ± SEM. **p < 0.01, ***p < 0.001. **c** Expression of SNX4 in AD (*n* = 8) and non-AD (*n* = 7) brains analyzed by Western blotting with antibodies against SNX4, BACE1, Aβ, and β-actin. Representative images and graphs are shown. Data are presented as mean ± SEM. **p < 0.01, ***p < 0.001

**Fig. 2:**
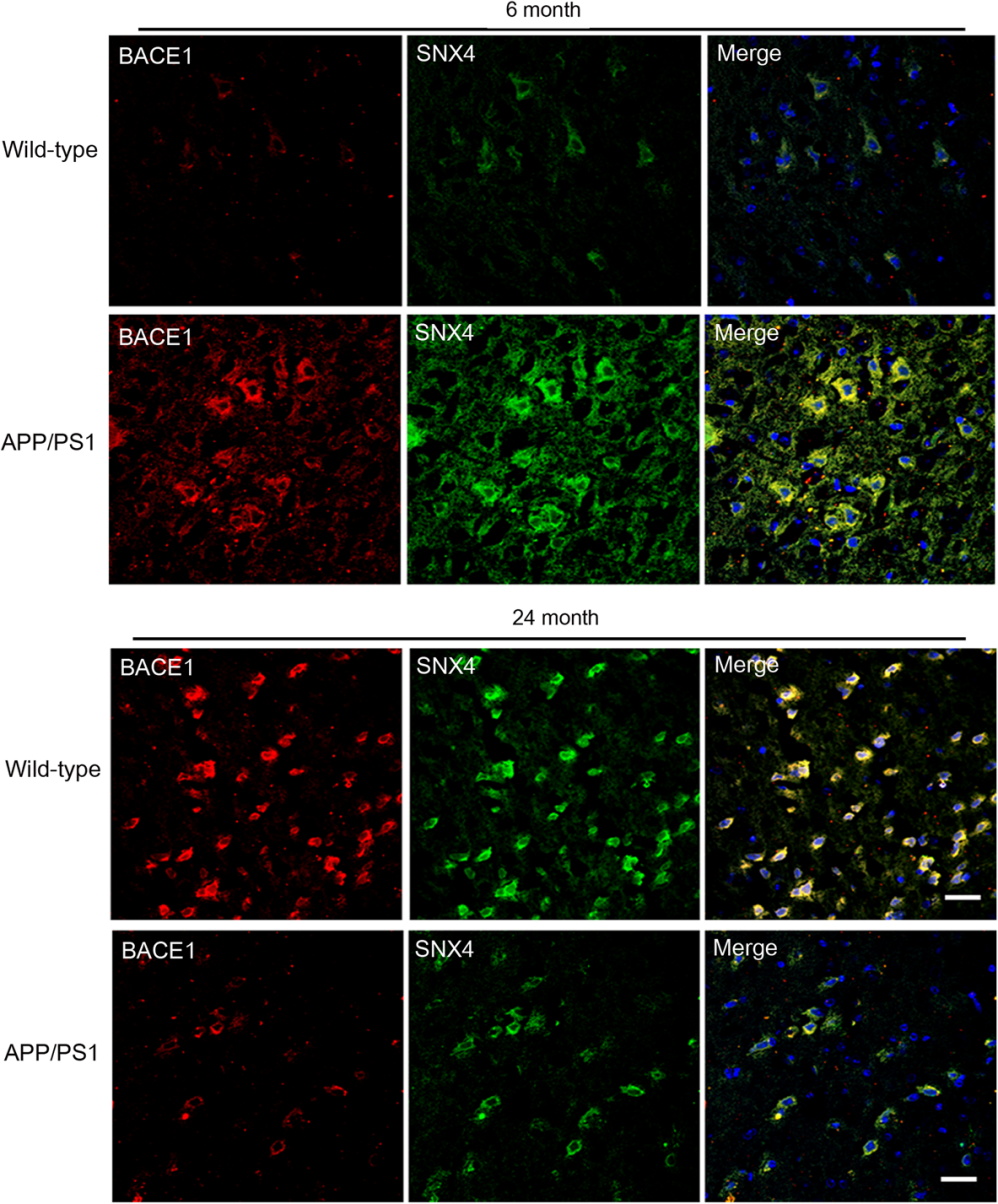
Altered levels of sorting nexin-4 (SNX4) and β-site amyloid precursor protein-cleaving enzyme 1 (BACE1) in the brains of APP/PS1 mice. Immunohistochemistry was performed using anti-BACE1 (*red*) and anti-SNX4 (*green*) antibodies in wild-type and APP/PS1 mice aged 6 months and 24 months. The intensity of BACE1 and SNX4 in neurons is increased in the cortices of 6-month-old APP/PS1 mice compared with wild-type mice. The intensity of BACE1 and SNX4 in neurons is decreased in the cortices of 24-month-old APP/PS1 mice compared with wild-type mice. Scale bar = 20 μm

**Fig. 3:**
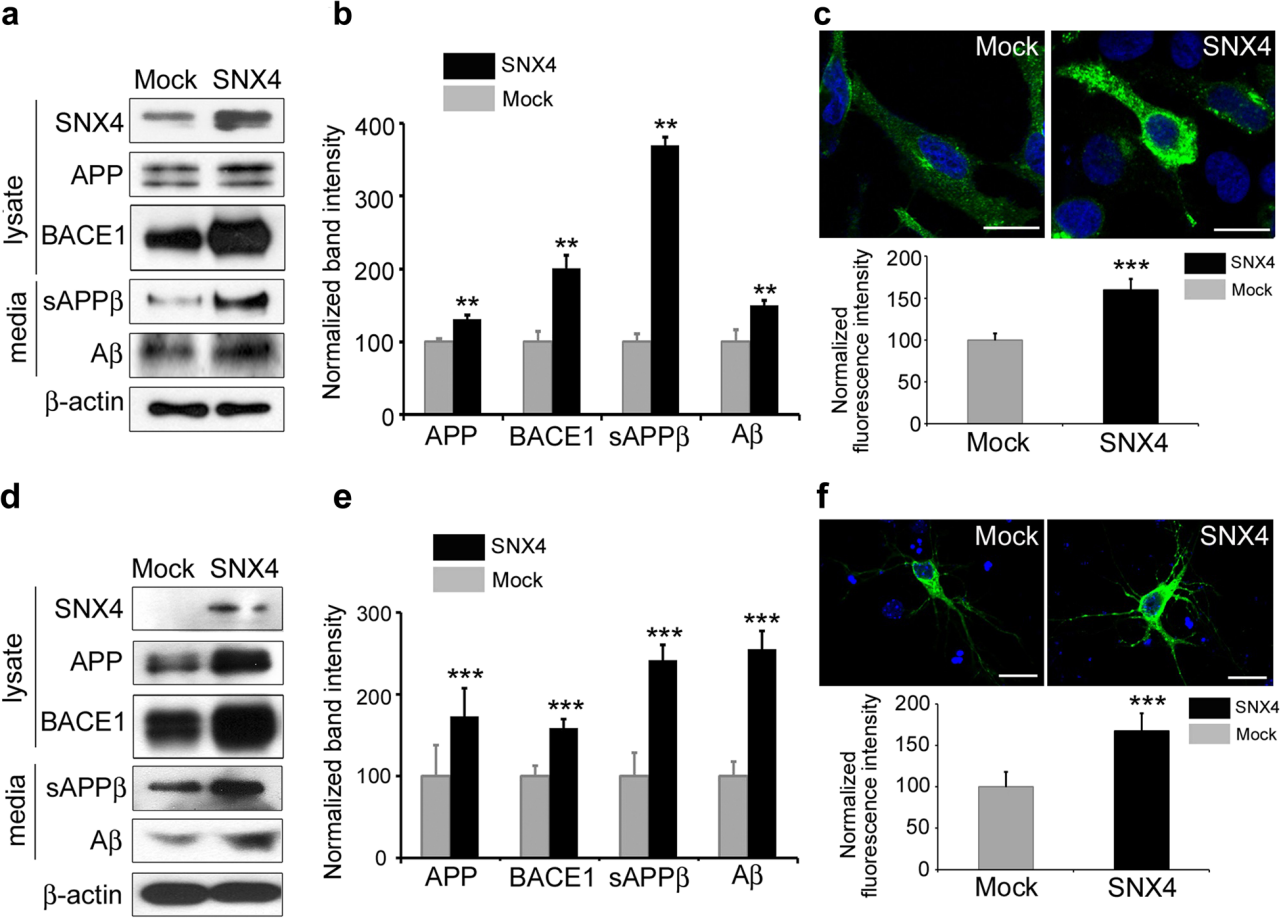
Sorting nexin-4 (*SNX4*) expression increases β-site amyloid precursor protein-cleaving enzyme 1 (BACE1) levels and β-amyloid (Aβ). **a** HEK293 cells were cotransfected with *BACE1* and either mock or *SNX4*. Quantitative Western blot analysis was performed using anti-BACE1, anti-β-actin, anti-Aβ (6E10), anti-amyloid precursor protein (anti-APP), anti-soluble ectodomain-secreted β-amyloid precursor protein derivative (anti-sAPPβ), and anti-SNX4 antibodies. The amount of Aβ was analyzed in the culture media by immunoblotting. **b** Quantification of Western blot band intensities. The graphs display the immunoreactivity to anti-BACE1, anti-APP, anti-sAPPβ, and anti-Aβ antibodies, normalized to β-actin. **p < 0.01, ***p < 0.001. **c** HeLa cells were cotransfected with *BACE1*-hemagglutinin (HA) and either mock or *SNX4*. Immunocytochemistry was performed using an anti-HA antibody (*green*). Scale bar = 20 μm. **d** Primary mouse cortical neurons were transfected with either mock green fluorescent protein (*GFP*) or *SNX4-GFP*. Quantitative Western blot analysis was performed using anti-BACE1, anti-Aβ (6E10), anti-APP, anti-sAPPβ, anti-β-actin, and anti-GFP antibodies. The amounts of Aβ and sAPPβ were analyzed in the culture medium by immunoblotting. **e** Quantification of Western blot band intensities. The graphs display the immunoreactivity to BACE1, APP, sAPPβ, and Aβ antibodies, normalized to β-actin. **p < 0.01, ***p < 0.001. **f** Primary neurons were cotransfected with *BACE1*-HA and either mock or *SNX4*. Immunocytochemistry was performed using an anti-HA antibody (*green*). Scale bar = 20 μm

To investigate whether SNX4 is involved in the pathogenesis of AD, we compared the levels of SNX4 protein between controls and patients with AD. The total level of SNX4 protein was approximately 70% less in late-stage AD brains than in controls (Fig. 1c). To investigate temporal changes of SNX4 levels during the aging process, we checked SNX4 levels using transgenic APP/PS1 AD model mice. Interestingly, SNX4 levels in the brains of 6-month-old APP/PS1 mice were increased compared with wild-type mice, whereas SNX4 levels were decreased in the brains of 24-month-old APP/PS1 mice compared with controls (Fig. 1, Additional file 1: Figure S1). We also performed immunohistochemistry using anti-BACE1 and anti-SNX4 in wild-type and APP/PS1 mouse brain tissue. Consistent with the Western blot analysis results, BACE1 and SNX4 levels in the brain tissue of 6-month-old APP/PS1 mice were increased compared with those of wild-type mice, whereas SNX4 levels were decreased and BACE1 levels were unchanged in the brain tissue of 24-month-old APP/PS1 mice and AD brain compared with controls (Figs. 2 and 3). BACE1 antibody specificity was verified additionally (Additional file 2: Figure S2).

### SNX4 increases BACE1 and β-processing of APP

Our observations of increased SNX4 levels in the brains of young APP/PS1 mice and decreased SNX4 levels in the brains of old APP/PS1 mice and patients with late-stage AD prompted us to investigate the role of SNX4 by overexpression and knockdown. To this end, HEK293 cells were cotransfected with BACE1 and SNX4 to examine if SNX4 affected Aβ generation. Levels of BACE1 were increased in SNX4-transfected cells compared with mock-transfected cells (Fig. 3a and b). sAPPβ and Aβ was also increased in the culture media by SNX4 overexpression (Fig. 3a and b). Next, HeLa cells were cotransfected with BACE1-HA and SNX4 and immunostained with HA antibody. In these experiments, BACE1 was increased in SNX4-transfected cells compared with mock-transfected cells (Fig. 3c). Primary mouse cortical neurons were also cotransfected with BACE1 and SNX4 to confirm the effects in neurons, and we found that levels of BACE1 and secreted sAPPβ and Aβ were increased in SNX4-transfected neurons compared with mock-transfected cells (Fig. 3d and e). Immunocytochemistry also showed that BACE1 was increased in SNX4-transfected neurons compared with mock-transfected neurons (Fig. 3f). These results show that SNX4 overexpression increases BACE1 levels and subsequently leads to an increase in the BACE1-mediated, APP-processing product Aβ.

### SNX4 knockdown leads to decreased BACE1 and β-processing of APP

**Fig. 4:**
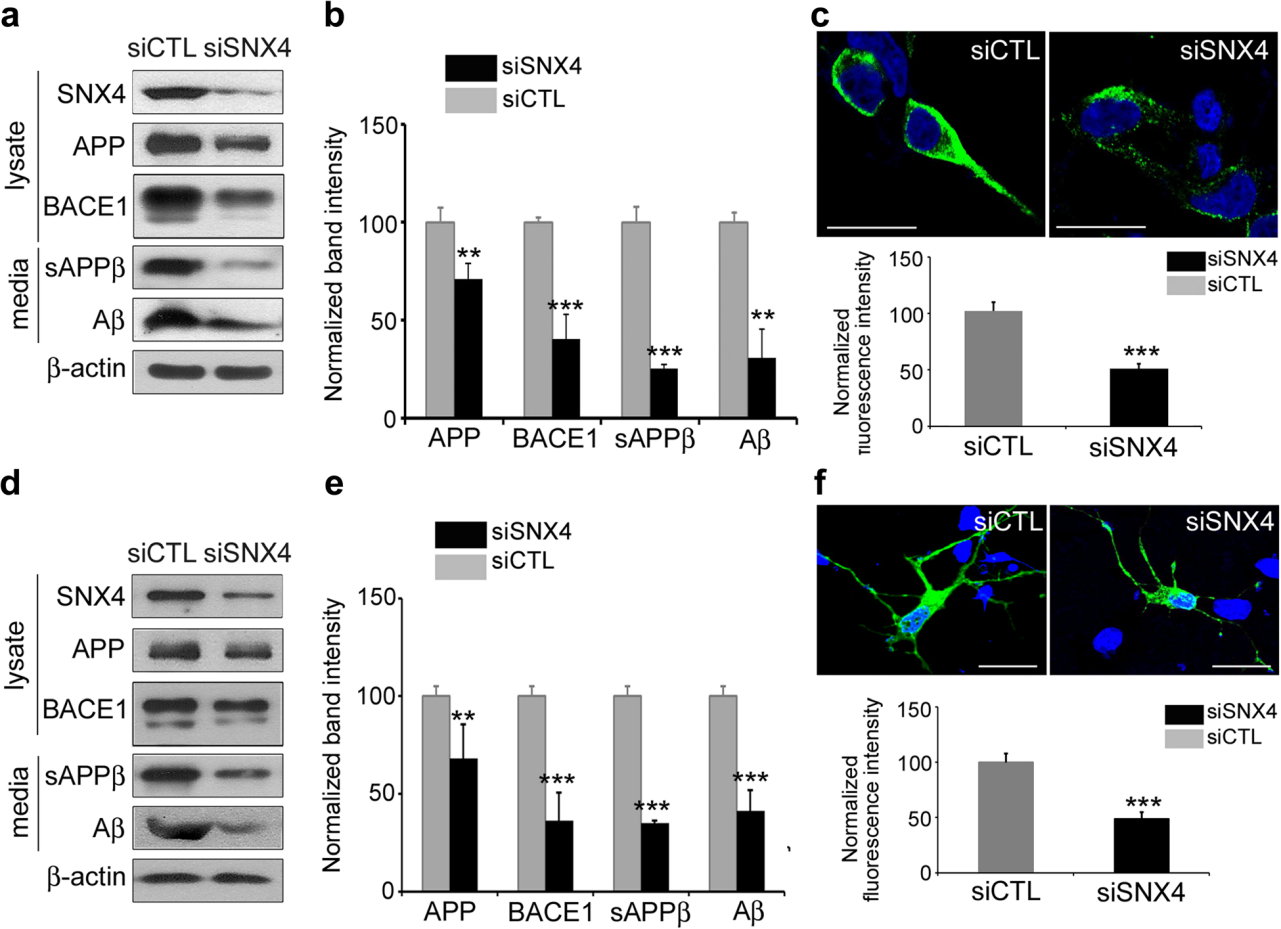
Reduced sorting nexin-4 (*SNX4*) levels decrease β-site amyloid precursor protein-cleaving enzyme 1 (BACE1) and β-amyloid (Aβ). **a** SH-SY5Y cells were cotransfected with *BACE1* and either *SNX4* small interfering RNA (siRNA) or control siRNA (siCTL). Quantitative Western blot analysis using anti-BACE1, anti-Aβ (6E10), anti-β-actin, anti-amyloid precursor protein (anti-APP), anti-soluble ectodomain-secreted β-amyloid precursor protein derivative (anti-sAPPβ), and anti-SNX4 antibodies was performed, and the amounts of Aβ and sAPPβ in the culture medium were analyzed by immunoblotting. **b** Quantification of Western blot band intensities. The graphs display the immunoreactivity to BACE1, APP, sAPPβ, and Aβ antibodies, normalized to β-actin. **p < 0.01, ***p < 0.001. **c** SH-SY5Y cells were transfected with either *siCTL* or *siSNX4*. Immunocytochemistry was performed using an anti-hemagglutinin (anti-HA) antibody (*green*). Scale bar = 20 μm. **d** Primary mouse cortical neurons were cotransfected with *BACE1* and either *shCTL* or *shSNX4*. Western blot analysis was performed using anti-BACE1, anti-APP, anti-sAPPβ, anti-β-actin, anti-Aβ (6E10), and anti-SNX4 antibodies. The amounts of Aβ and sAPPβ were analyzed in the culture medium by immunoblotting. **e** Quantification of Western blot band intensities. The graphs display the immunoreactivity to anti-BACE1, anti-APP, anti-sAPPβ, and anti-Aβ antibodies, normalized to β-actin. **p < 0.01, ***p < 0.001. **f** Primary neurons were cotransfected with *BACE1*-HA and either *shCTL* or *shSNX4*. Immunocytochemistry was performed using an anti-HA antibody (*green*). Scale bar = 20 μm

The knockdown of SNX4 with a siRNA mixture decreased the level of BACE1 in SH-SY5Y cells and mouse cortical neurons (Fig. 4a and b). The levels of sAPPβ and Aβ in the culture media was also decreased following an SNX4 knockdown (Fig. 4a and b). SH-SY5Y cells were transfected with BACE1-HA and either siCTL or siSNX4, then immunostained with HA antibody. BACE1 was downregulated in siSNX4-transfected cells compared with siCTL-transfected cells (Fig. 4c). Primary mouse cortical neurons were also transfected with BACE1 and either siCTL or siSNX4 to confirm the effects of SNX4 in neurons. Levels of BACE1 and secreted Aβ were decreased in siSNX4-transfected neurons compared with siCTL transfected cells (Fig. 4d and e). Immunocytochemistry also revealed that BACE1 was decreased in siSNX4-transfected neurons compared with siCTL-transfected neurons (Fig. 4f). The results obtained with siRNAs were confirmed with various target sequences and recovery experiments (Additional file 3: Figure S3). These results indicated that SNX4 knockdown downregulated BACE1 levels and subsequently led to decreased levels of the BACE1-mediated, APP-processing product Aβ.

### SNX4 interacts with BACE1

**Fig. 5:**
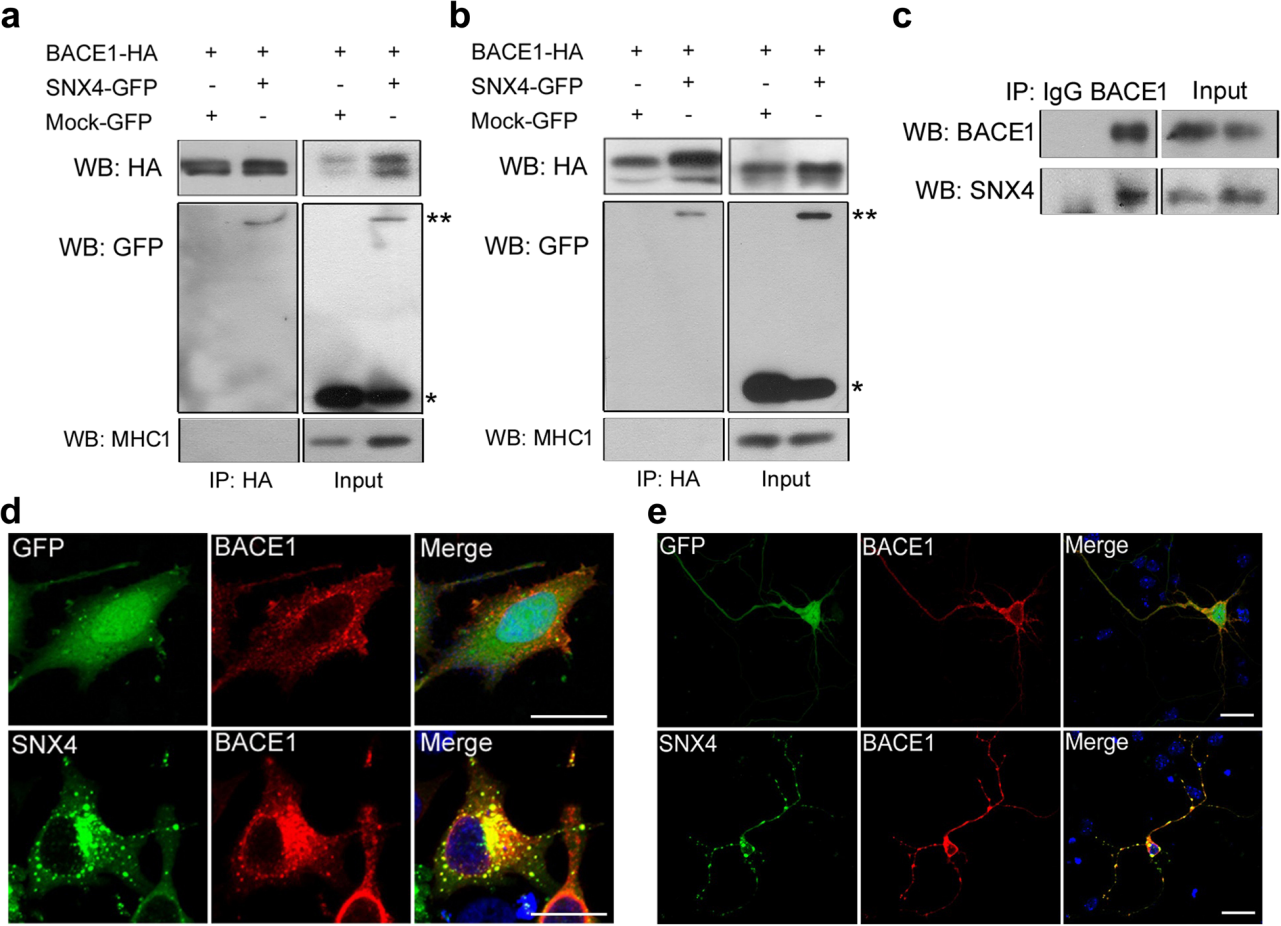
Sorting nexin-4 (SNX4) interacts with β-site amyloid precursor protein-cleaving enzyme 1 (BACE1). **a** HEK293 cells were cotransfected with *BACE1-*hemagglutinin (*BACE1-HA*) and either mock green fluorescent protein (mock-*GFP*) or *SNX4-GFP*. HEK293 cell lysates were coimmunoprecipitated (IP) with HA antibodies, followed by Western blotting (WB) against HA, GFP, and major histocompatibility complex class I (MHC1) antibodies to assess their interaction. **b** Mouse cortical neurons were cotransfected with *BACE1-HA* and either mock-*GFP* or *SNX4-GFP*. Cell lysates were coimmunoprecipitated with HA antibodies, followed by Western blotting against HA, GFP, and MHC1 antibodies to assess their interaction. **c** Mouse brain lysates were coimmunoprecipitated with BACE1 antibodies, followed by Western blotting against BACE1 or SNX4 antibodies to assess their endogenous interaction. **d** HeLa cells were cotransfected with *BACE1*-*HA* and either mock-*GFP* or *SNX4-GFP*. Immunocytochemistry was performed using an anti-HA antibody (*red*). Scale bar = 20 μm. **e** Cortical neurons were cotransfected with *BACE1*-*HA* and either mock-*GFP* or *SNX4-GFP*. Immunocytochemistry was performed using an anti-HA antibody (*red*). Scale bar = 20 μm

To determine whether SNX4 modulated BACE1-mediated APP processing/Aβ generation through a direct interaction, we carried out coimmunoprecipitation between SNX4 and BACE1 in HEK293 cells and mouse cortical neurons transfected with BACE1-HA and either mock-GFP or SNX4-GFP. We found that an anti-HA antibody pulled down SNX4-GFP but not the mock-GFP. The specificity of coimmunoprecipitation was confirmed through immunoblot analysis using anti-MHC-1 antibody, showing no interaction between BACE1 and another membrane protein, MHC-1 (Fig. 5a, b). We further carried out coimmunoprecipitation between endogenous SNX4 and BACE1 in mouse brain tissue and found that an anti-BACE1 antibody pulled down SNX4 (Fig. 5c). Immunocytochemistry with an HA antibody also showed that SNX4-GFP and BACE1-HA colocalized in HeLa cells and primary neurons (Fig. 5d, e). In contrast to BACE1, presenilin-1 does not interact with SNX4 (Additional file 4: Figure S4).

### SNX4 shifts BACE1 from the degradation pathway and increases its half-life

**Fig. 6:**
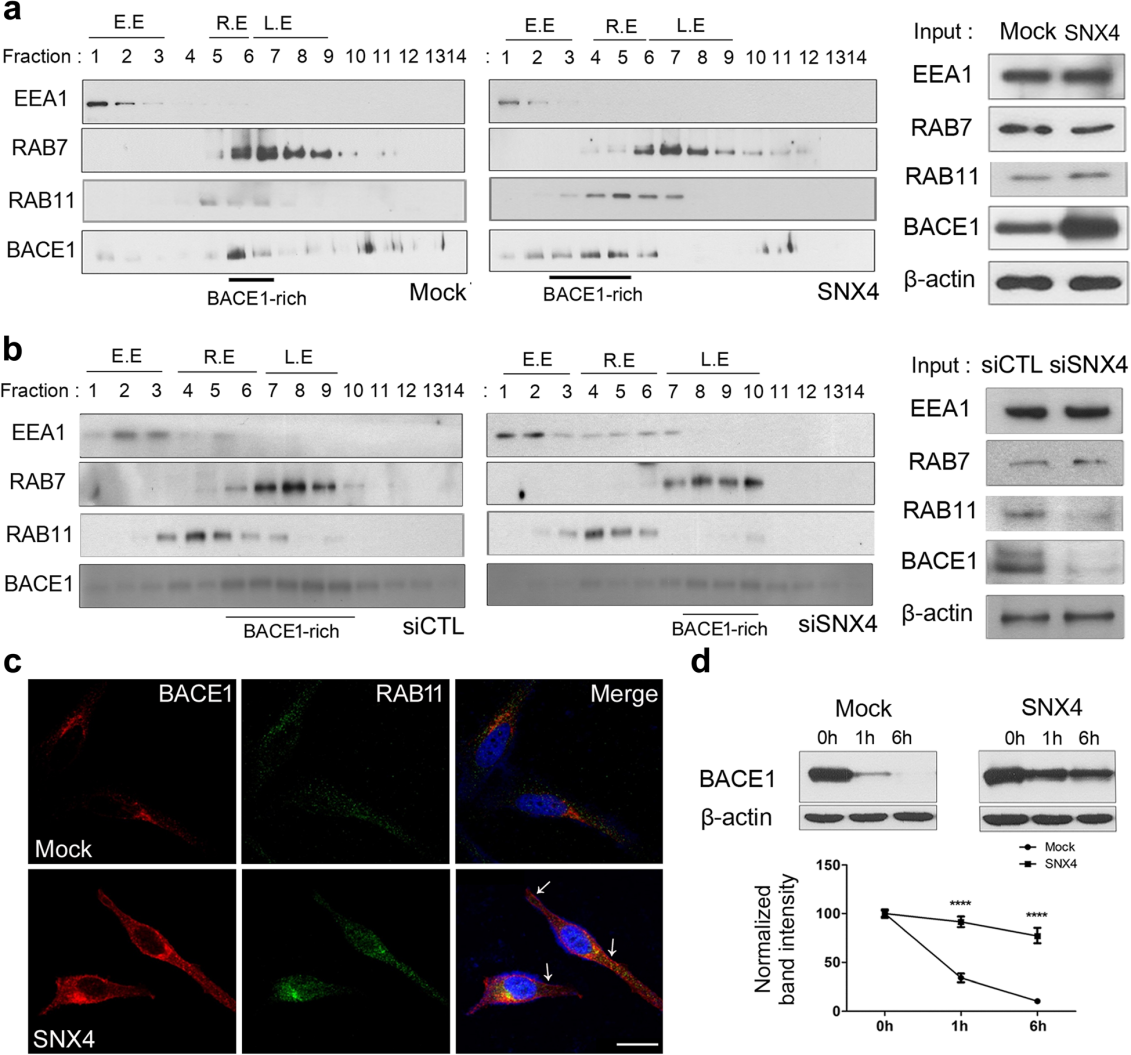
Sorting nexin-4 (SNX4) shifts β-site amyloid precursor protein-cleaving enzyme 1 (BACE1) away from the degradation pathway. **a** Gradient fractionation was performed on SH-SY5Y cells cotransfected with *BACE1* and either mock or *SNX4* constructs. In mock-transfected cells, strong BACE1 signals appeared in fractions 6 and 7, which overlap with Rab7, a late endosome marker. BACE1 appeared primarily in fractions 3–5, which overlap more with Rab11, a recycling endosome marker. E.E., early endosome; R.E., recycling endosome; L.E., late endosome. **b** Gradient fractionation was performed on SH-SY5Y cells cotransfected with *BACE1* and either scrambled small interfering RNA (*siCTL*) or small interfering RNA mixture (*siSNX4*). In *siCTL*-transfected cells, strong BACE1 signals appeared in fractions 6–10, which overlap with Rab7 and Rab11. BACE1 appeared primarily in fractions 7–10, which overlap more with Rab7. **c** Immunocytochemistry was performed using an anti-BACE1 antibody (*red*) and anti-Rab11 antibody (*green*) in SH-SY5Y cells. *Arrows* indicate BACE1 localization to the cell membrane. Scale bar = 20 μm. **d** HEK293 cells expressing BACE1 were transfected with mock or *SNX4* constructs and cotreated with cycloheximide, a protein synthesis inhibitor. BACE1 decreased very rapidly after 1 h in mock-transfected cells but decreased slowly in *SNX4*-transfected cells. ****p < 0.001. EEA1, Early endosome antigen 1

Given our finding that SNX4 interacts with BACE1 and regulates its levels, we investigated the effects of SNX4 on BACE1 trafficking and turnover. Gradient fractionation was performed in SH-SY5Y cells. In control cells, BACE1 was found to be localized broadly through all fractions but was especially abundant in fractions 6 and 7 (Fig. 6a), which overlapped with Rab7, a late endosome marker, suggesting that a significant portion of BACE1 traffics to the degradation pathway. Interestingly, in SNX4-transfected cells, the primary localization of BACE1 shifted to fractions 3–5 (Fig. 6a), which overlapped more with Rab11, a recycling endosome marker. The knockdown of SNX4 narrowed the range of BACE1-rich fractions and shifted the fractions to Rab7-positives, whereas BACE1-rich fractions were broad, including either Rab11- and Rab7-positives in siCTL-transfected cells (Fig. 6b). These results suggested that BACE1 had escaped from the degradation pathway, probably via SNX4-dependent effects on its recycling. Immunocytochemistry also showed that BACE1 was colocalized in Rab11 and trafficked to the surface in SNX4-transfected SH-SY5Y (Fig. 6c, *arrows*). The half-life of BACE1 was tested in HEK293 cells expressing BACE1 using the protein synthesis inhibitor cycloheximide. In control cells, BACE1 decreased very rapidly after cycloheximide treatment (Fig. 6d), indicating that BACE1 is rapidly degraded under normal conditions. However, in SNX4-transfected cells, BACE1 decreased slowly after cycloheximide treatment compared with controls (Fig. 6d), suggesting that SNX4 may protect BACE1 from the degradation pathway (Fig. 8).

### SNX4 regulates cell membrane levels of BACE1

**Fig. 7:**
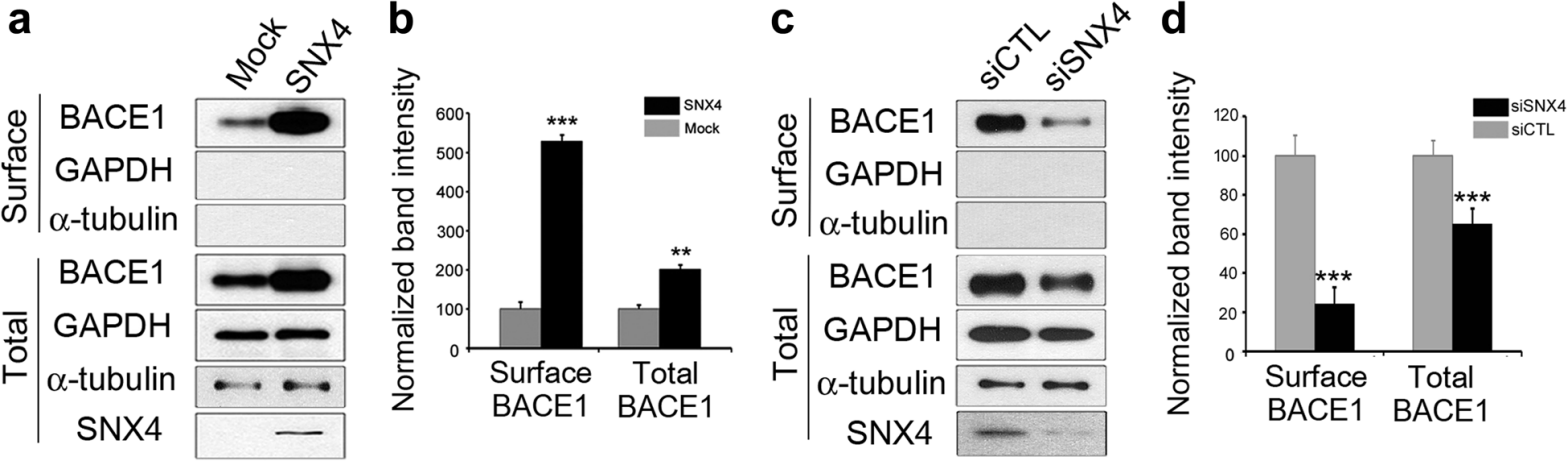
Sorting nexin-4 (SNX4) increases the plasma membrane levels of β-site amyloid precursor protein-cleaving enzyme 1 (BACE1). **a**, **c** HEK293 cells were transiently transfected with *SNX4* and *BACE1*, and SH-SY5Y cells were transfected with *siSNX4* and *BACE1*. BACE1 was biotinylated, and the expression levels of total and cell surface BACE1 were analyzed by quantitative Western blotting using anti-BACE1 antibodies. Glyceraldehyde 3-phosphate dehydrogenase (GAPDH) and α-tubulin in the surface fraction was used as a negative control to confirm fractionation. **b**, **d** Western blot bands were quantitated, and graphs indicating the immunoreactivity to the BACE1 antibody, normalized to GAPDH, are shown. **p < 0.01, ***p < 0.005

Because our results suggest that SNX4 protects BACE1 from the degradation pathway and increases its half-life (Fig. 6), we speculated that SNX4 may be involved in the recycling of BACE1. Hence, we carried out a cell surface protein biotinylation assay for BACE1. Upon overexpression of SNX4, the levels of cell surface BACE1 as well as total BACE1 increased, whereas these levels decreased when SNX4 was knocked down compared with each control (Fig. 7a, c). GAPDH and α-tubulin in the cell surface fraction were used as a negative control for accurate fractionation. Upon SNX4 expression, total BACE1 levels increased 2.8-fold, and the levels of BACE1 at the cell surface increased 5.6-fold (Fig. 7b). Knockdown of SNX4 decreased total BACE1 levels by 40% and surface BACE1 levels by 20% (Fig. 7d).

## Discussion

In our present study, we found that SNX4 levels are altered in the brains of patients with AD and in AD model mice. Our data reveal that SNX4 interacts with BACE1 and increases its steady-state levels, leading to increased generation of Aβ. Our results also indicate that SNX4 enhances the recycling of BACE1 from sorting endosomes to the plasma membrane. This recycling of BACE1 by SNX4 prevents the trafficking of BACE1 to late endosomes and lysosomes for degradation, increasing the half-life of BACE1 and β-processing of APP.

SNX4 is a member of the PX domain-containing trafficking molecule family involved in membrane trafficking [19, 20]. One feature of SNXs is their ability to support cargo complex formation by binding specific lipids and aiding donor membrane curvature via their PX and BAR domains [28, 29]. Another role of SNXs is to tightly control the levels of their selected target cargo proteins in a given organelle. SNX4 localizes to Rab11^+^ recycling endosomes, which have abundant SNX4 but relatively low levels of SNX1 and SNX8 [20]. SNX4 is also involved in the recycling of the transferrin receptor. Sorting tubules are formed by SNX4 from Rab4^+^/Rab11^+^ endosomes, indicating that recycling endosomes are extended by SNX4-dependent membrane trafficking, whereas SNX1 or SNX8 associates with TGN-targeted tubules in the early-to-late endosome pathway [20] Collectively, SNX4 may regulate the recycling of specific cargoes to the plasma membrane. This is supported by our finding that SNX4 recycles BACE1 to the plasma membrane and protects it from the degradation pathway (Figs. 6, and 7).

**Fig. 8:**
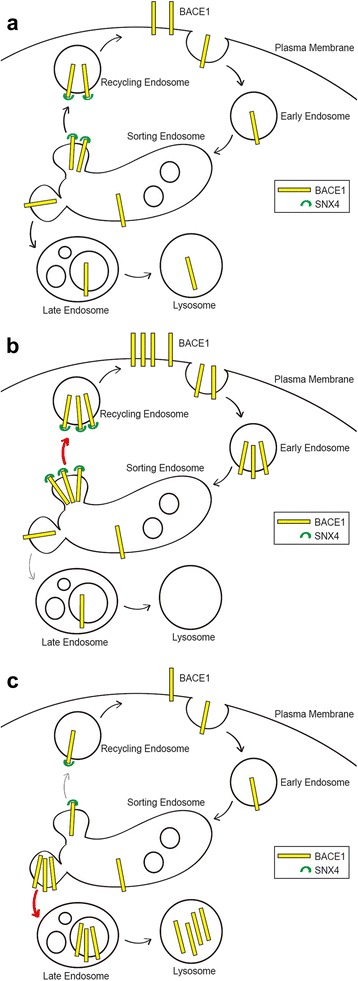
Putative models of the role of sorting nexin-4 (SNX4) role in β-site amyloid precursor protein-cleaving enzyme 1 (BACE1) trafficking. **a** BACE1 in the plasma membrane is endocytosed and trafficked to the sorting endosomes. BACE1 is then sorted to the late endosomes and lysosomes for degradation. If BACE1 interacts with SNX4 during the sorting processes, it is recycled to the plasma membrane by SNX4 and shifted away from the lysosomal degradation pathway, resulting in a longer half-life and more chances to generate β-amyloid. **b** When SNX4 is upregulated, such as in the early stage in Alzheimer’s disease model mice, SNX4 recycles BACE1 and prevents its degradation. **c** When SNX4 is downregulated, the recycling of BACE1 is decreased, and its degradation increases

We first hypothesized that decreased SNX4 might induce Aβ generation and pathology because we observed decreased SNX4 levels in AD brains (Figs. 1c, and 2). However, our experiments using overexpression and knockdown of SNX4 revealed opposite results (Figs. 3, and 4). Our AD brain samples represented late-stage disease (Braak stages 5 and 6) (Table 1). In the brain samples of AD model mice, SNX4 levels increased at age 6 months but decreased at age 24 months (Figs. 1a and b, 2). Taken together, these results suggested that SNX4 might be upregulated in the early stages of AD pathogenesis and might augment Aβ generation by regulating the recycling of BACE1 and preventing it from lysosomal degradation (Fig. 8). However, SNX4 is downregulated in the late stages of AD by unidentified mechanisms. This downregulation might be a compensatory response to inhibit Aβ generation, or it might represent a by-product of neurodegeneration. These possibilities can be addressed in future studies. Upregulation of SNX4 in the early stages of AD is an important issue because therapeutic reduction of SNX4 can inhibit generation of Aβ. Hence, future studies are necessary to address the mechanism of SNX4 upregulation in the early stages of AD.

The critical and initial point of Aβ generation is mediated by BACE1; hence, it is very important to understand which molecular machinery regulates the trafficking of BACE1, affecting Aβ generation. Although we found the direct interaction of BACE1 and SNX4 (Fig. 5) that accounts for changes in BACE1-mediated APP processing (Fig. 3, and 4), APP processing could also rely on the APP level itself. SNX4 also regulated the APP full-length levels (Fig. 3, and 4), which could affect BACE1-mediated APP processing. We think that SNX4 could regulate both APP and BACE1, affecting Aβ levels by the direct interaction of BACE1 and SNX4 and the indirect and unknown mechanism of APP. BACE1 has been shown to transit through the secretory pathway and target the endosomal system, cycling between endosomes and the cell surface, probably via the TGN [10, 11]. In addition to our findings of SNX4’s roles in BACE1 trafficking, several molecular mechanisms of regulating BACE1 have been reported. The endosomal trafficking of BACE1 appears to be partially regulated by an acidic cluster-dileucine motif in its cytoplasmic tail [30–32]. This motif has been shown to interact with the Vps27/Hrs/signal-transducing adapter molecule domain of Golgi-localized γ-adaptin ear-containing ADP-ribosylation factor-binding (GGA) proteins GGA1, GGA2, and GGA3, adapter proteins that mediate sorting between the TGN and endosomes [31, 33]. Recently, GGA3 was shown to bind BACE1 via the ubiquitin-sorting machinery and to regulate BACE1 degradation [34]. Although we showed that BACE1 level is regulated by SNX4, BACE1 was not decreased in the brains of 24-month-old mice or in late-stage AD brains (Fig. 1, and 2). This phenomenon let us analogize that BACE1 escaping from the recycling pathway may be not degraded, despite a decrease of SNX4 due to other factors, including inefficiency of lysosomal degradation or the endocytic pathway, as observed in AD-like pathological conditions [35, 36]. Decreased BACE1 levels were protected by inhibiting lysosomal acidification or endocytosis using bafilomycin A1 or chlorpromazine in siSNX4-transfected cells (Additional file 5: Figure S5), which may explain how BACE1 is not decreased in the brains of 24-month-old mice or in late-stage AD brains. Although it has been reported that BACE1 levels were increased in patients with AD [37], some studies have shown that BACE1 levels were not elevated in AD temporal cortex [38], in line with our present results.

## Conclusions

Our data indicate that SNX4-mediated regulation of the steady-state levels and trafficking of BACE1, as well as the subsequent increase in BACE1-mediated cleavage, may be relevant to AD progression. Regulating the expression levels of BACE1 to reduce Aβ production remains a promising strategy for therapeutic intervention in AD. Inhibition of BACE1 expression by strategies such as SNX4 modulation may be a critical strategy in developing AD therapeutics.

## Additional files

Additional file 1: Figure S1. The original immunoblots of represented proteins in Fig. 1 in the main text. (JPG 882 kb)
Additional file 2: Figure S2. BACE1 antibody specifically detects BACE1 in immunoblot analysis. HeLa cells were transfected with GFP, GFP-BACE1, HA, or HA-BACE1, and expression of BACE1 in each cell was analyzed by immunoblotting using anti-BACE1 antibody. The bands were detected in only GFP-BACE1- or HA-BACE1-transfected cells and appeared at close to the appropriate size marker (GFP-BACE1 approximately 100 kDa, HA-BACE1 approximately 75 kDa). (JPG 127 kb)
Additional file 3: Figure S3. SNX4 does not interact with presenilin-1 and control APLP2 processing. **a** SH-SY5Y cells were transfected with *BACE1*-HA and either mock *GFP* or *SNX4*. The cell lysates were coimmunoprecipitated with GFP antibody followed by Western blotting against anti-GFP and anti-presenilin-1 antibodies to assess interaction between SNX4 and presenilin-1. **b** SH-SY5Y cells were transfected with *BACE1*-HA and either mock-*GFP* or *SNX4*, and the levels of APLP2 were analyzed by immunoblotting in either cell lysates or culture medium. The bar graph shows the band densities of the sAPLP2 in medium as a percentage of the indicated group. Data are presented as mean ± SEM of three independent experiments and were analyzed using Student’s *t* test. ***p* < 0.01, ****p* < 0.001 vs. control. (JPG 218 kb)
Additional file 4: Figure S4. The decrease of BACE1 is protected by inhibiting lysosomal acidification or endocytosis. SH-SY5Y cells were transfected with *BACE1*-HA and either mock-*GFP or SNX4* and incubated with or without chlorpromazine (15 μM) or bafilomycin A1 (nM) for 24 h. The BACE1 levels were analyzed by immunoblotting in the cell lysates. The bar graph shows the band densities of BACE1 as a percentage of the indicated group. Data are presented as mean ± SEM of three independent experiments and were analyzed using Student’s *t* test. ***p* < 0.01, ****p* < 0.001 vs. control. (JPG 146 kb)
Additional file 5: Figure S5.
**a** SNX4 is sufficiently downregulated by siRNA. SH-SY5Y cells and mouse primary cortical neurons (DIV 5) were transfected with *siCTL* or three different targeting *siSNX4* siRNAs. The levels of SNX4 and BACE1 were analyzed by immunoblotting in the cell lysates. The siSNX4 siRNAs sufficiently decreased the levels of SNX4 and BACE1 compared with siCTL. **b** Mouse primary cortical neurons (DIV 5) were cotransfected with *BACE1*-HA and *siCTL*, *siSNX4* mixture, or *siSNX4* mixture and *SNX4*. The levels of SNX4, BACE1, APP, sAPPβ, and Aβ were analyzed by immunoblotting in the cell lysates. siSNX4 sufficiently decreased the levels of SNX4, BACE1, APP, and BACE1-mediated APP-processing products compared with siCTL, and the decrease of indicated protein levels was rescued with SNX4. **c** Quantification of Western blot band intensities. The graphs display the immunoreactivity to BACE1, APP, sAPPβ, and Aβ antibodies, normalized to β-actin (***p* < 0.01, ****p* < 0.001). **d** Primary neurons were cotransfected with *BACE1*-HA and *siCTL*, *siSNX4* mixture, or *siSNX4* mixture and *SNX4*. Immunocytochemistry was performed using an anti-HA antibody (*green*). Scale bar = 10 μm. (JPG 530 kb)

## Abbreviations

AD: Alzheimer’s disease
APP: Amyloid precursor protein
Aβ: β-Amyloid
BACE1: β-Site amyloid precursor protein-cleaving enzyme 1
CTF: C-terminal fragment
EDTA: Ethylenediaminetetraacetic acid
E.E.: Early endoeoms
EEA1: Early endosome antigen 1
GAPDH: Glyceraldehyde 3-phosphate dehydrogenase
GFP: Green fluorescent protein
GGA: Golgi-localized γ-adaptin ear-containing ADP-ribosylation factor-binding protein
HA: Hemagglutinin
L.E.: Late endosome
MHC1: Major histocompatibility complex class I
PBST: PBS containing 0.1% vol/vol Tween-20
PMD: Postmortem delay
PX: Phospholipid-binding motif
R.E.: Recycling endosome
RT: Room temperature
sAPPβ: Soluble ectodomain-secreted β-amyloid precursor protein derivative
siCTL: Scrambled small interfering RNA
siRNA: Small interfering RNA
siSNX4: Small interfering RNA mixture
SNX: Sorting nexin
SNX4: Sorting nexin-4
SNX-BAR: SNX protein containing a Bin/amphiphysin/Rvs domain
TGN: *Trans*-Golgi network
VPS: Vacuolar protein sorting
WB: Western blotting
WT: Wild type
