# Supplementary figures and images for: Sorting nexin-4 regulates β-amyloid production by modulating β-site-activating cleavage enzyme-1
Source: Alzheimers Res Ther. 2017 Jan 21;9:4. doi: 10.1186/s13195-016-0232-8 (PMC5251330; doi:10.1186/s13195-016-0232-8)

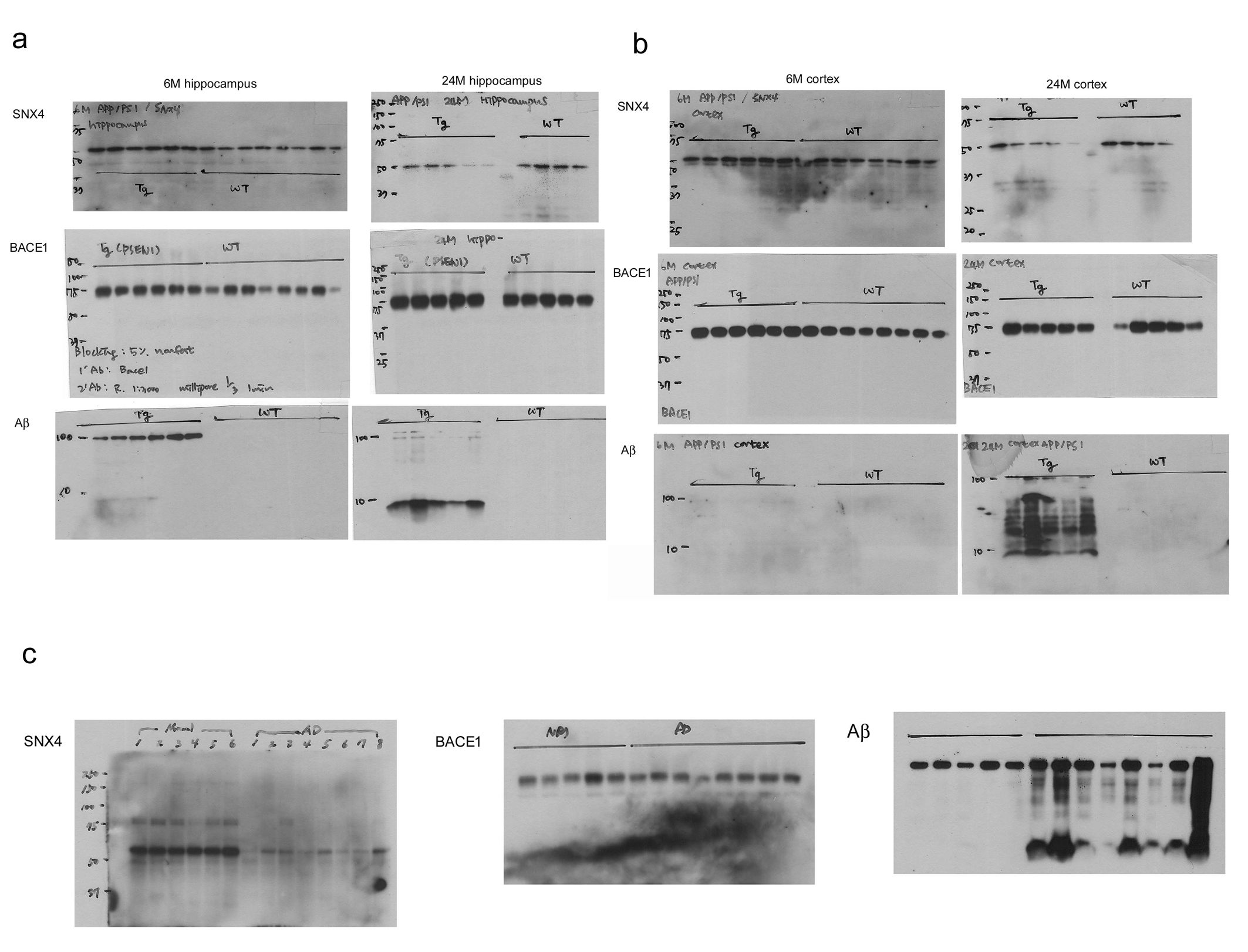

Supplement: Additional file 1: Figure S1. — The original immunoblots of represented proteins in Fig. 1 in the main text. (JPG 882 kb) [file 13195_2016_232_MOESM1_ESM.jpg]

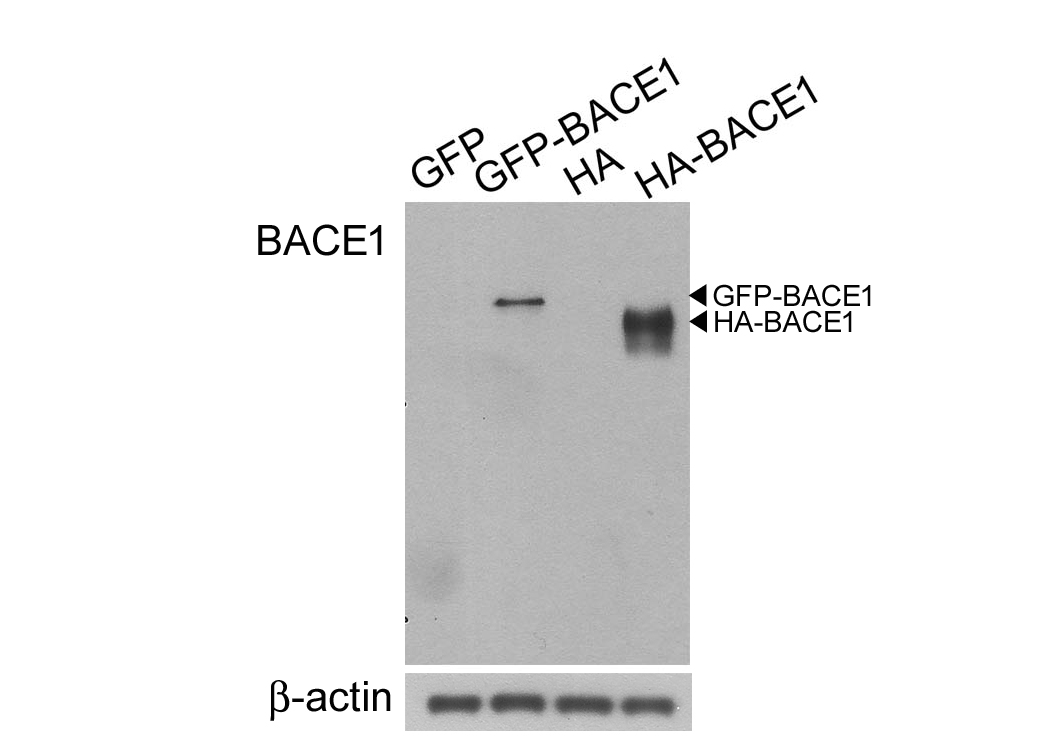

Supplement: Additional file 2: Figure S2. — BACE1 antibody specifically detects BACE1 in immunoblot analysis. HeLa cells were transfected with GFP, GFP-BACE1, HA, or HA-BACE1, and expression of BACE1 in each cell was analyzed by immunoblotting using anti-BACE1 antibody. The bands were detected in only GFP-BACE1- or HA-BACE1-transfected cells and appeared at close to the appropriate size marker (GFP-BACE1 approximately 100 kDa, HA-BACE1 approximately 75 kDa). (JPG 127 kb) [file 13195_2016_232_MOESM2_ESM.jpg]

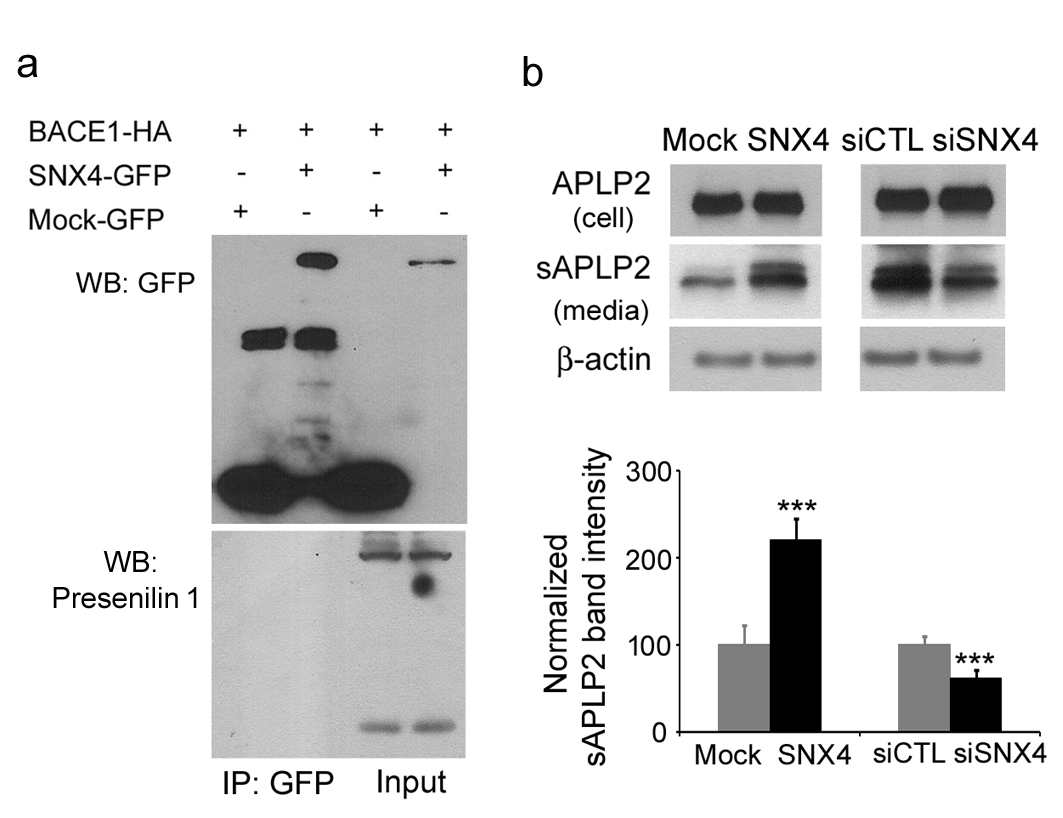

Supplement: Additional file 3: Figure S3. — SNX4 does not interact with presenilin-1 and control APLP2 processing. a SH-SY5Y cells were transfected with BACE1-HA and either mock GFP or SNX4. The cell lysates were coimmunoprecipitated with GFP antibody followed by Western blotting against anti-GFP and anti-presenilin-1 antibodies to assess interaction between SNX4 and presenilin-1. b SH-SY5Y cells were transfected with BACE1-HA and either mock-GFP or SNX4, and the levels of APLP2 were analyzed by immunoblotting in either cell lysates or culture medium. The bar graph shows the band densities of the sAPLP2 in medium as a percentage of the indicated group. Data are presented as mean ± SEM of three independent experiments and were analyzed using Student’s t test. **p < 0.01, ***p < 0.001 vs. control. (JPG 218 kb) [file 13195_2016_232_MOESM3_ESM.jpg]

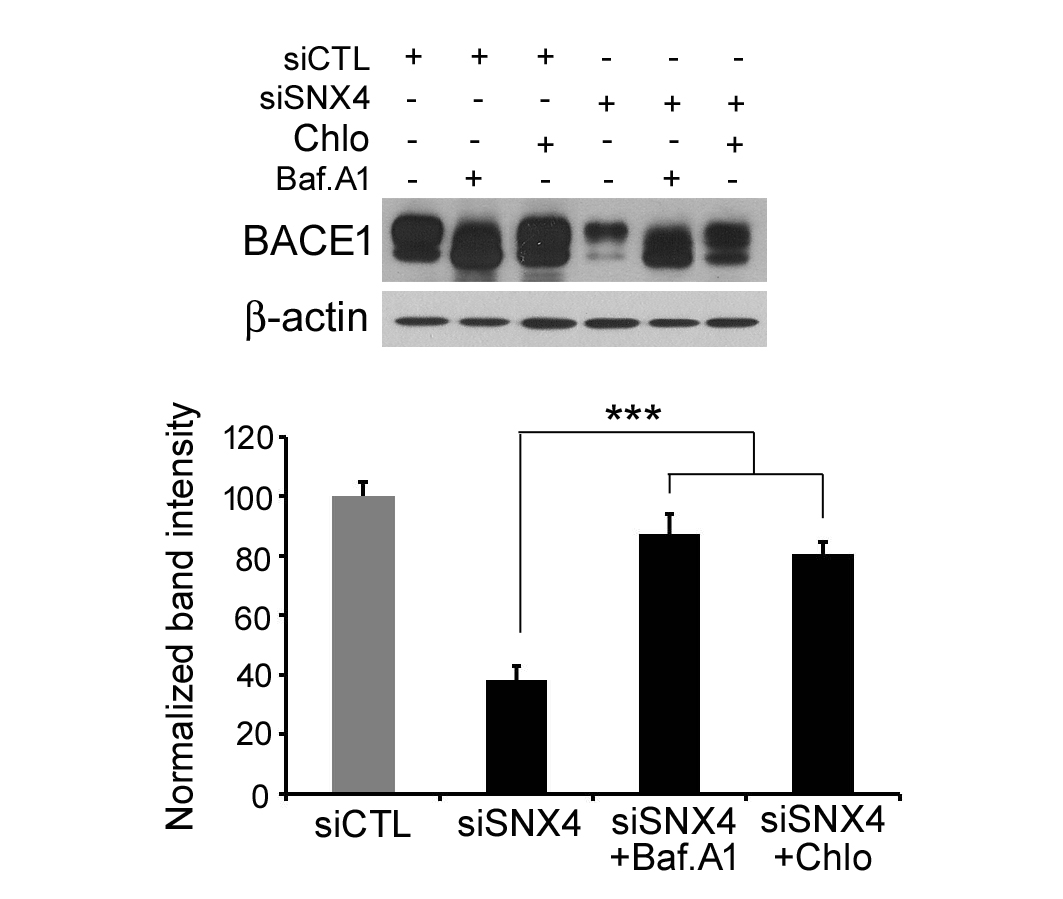

Supplement: Additional file 4: Figure S4. — The decrease of BACE1 is protected by inhibiting lysosomal acidification or endocytosis. SH-SY5Y cells were transfected with BACE1-HA and either mock-GFP or SNX4 and incubated with or without chlorpromazine (15 μM) or bafilomycin A1 (nM) for 24 h. The BACE1 levels were analyzed by immunoblotting in the cell lysates. The bar graph shows the band densities of BACE1 as a percentage of the indicated group. Data are presented as mean ± SEM of three independent experiments and were analyzed using Student’s t test. **p < 0.01, ***p < 0.001 vs. control. (JPG 146 kb) [file 13195_2016_232_MOESM4_ESM.jpg]

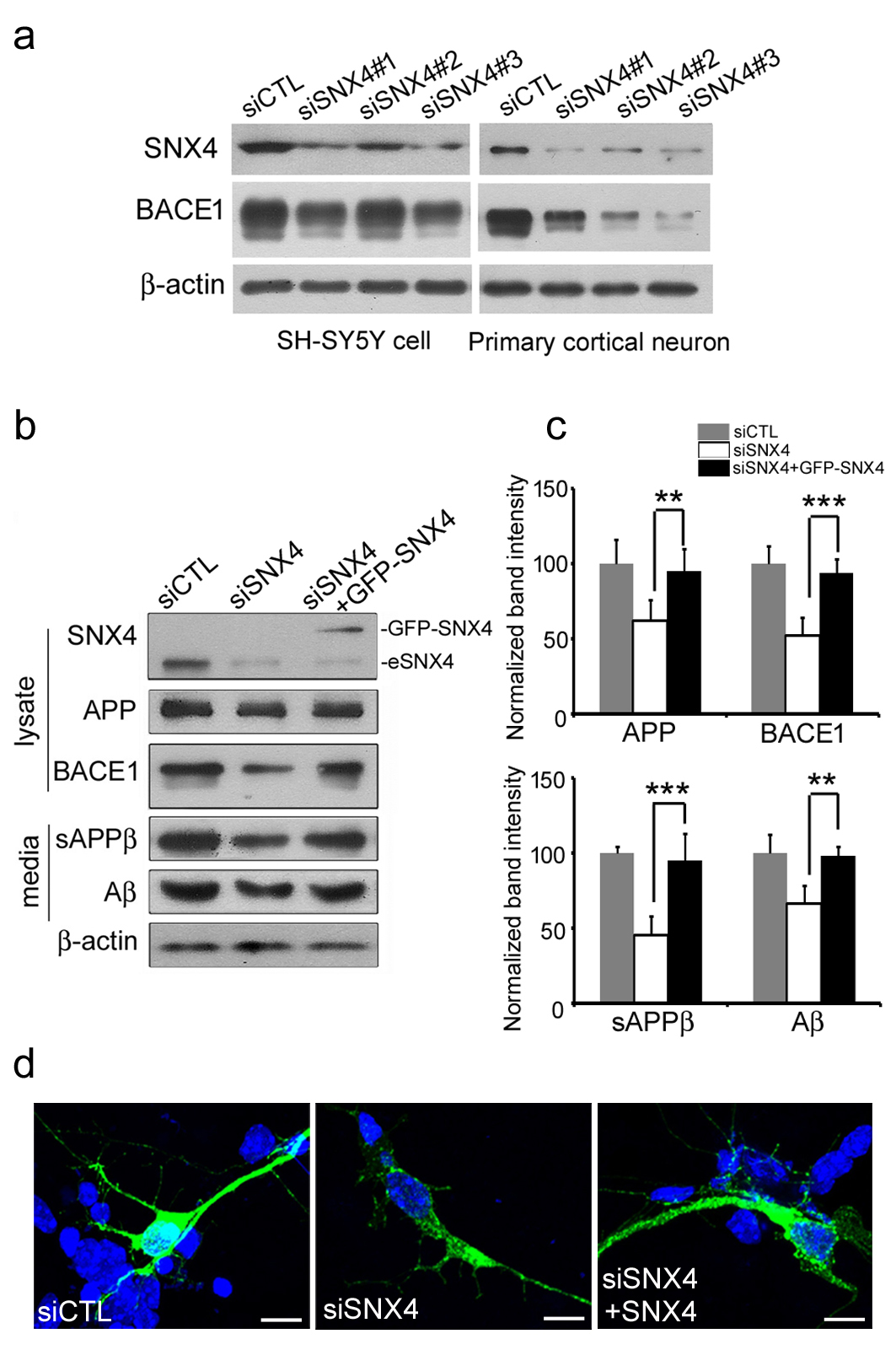

Supplement: Additional file 5: Figure S5. — a SNX4 is sufficiently downregulated by siRNA. SH-SY5Y cells and mouse primary cortical neurons (DIV 5) were transfected with siCTL or three different targeting siSNX4 siRNAs. The levels of SNX4 and BACE1 were analyzed by immunoblotting in the cell lysates. The siSNX4 siRNAs sufficiently decreased the levels of SNX4 and BACE1 compared with siCTL. b Mouse primary cortical neurons (DIV 5) were cotransfected with BACE1-HA and siCTL, siSNX4 mixture, or siSNX4 mixture and SNX4. The levels of SNX4, BACE1, APP, sAPPβ, and Aβ were analyzed by immunoblotting in the cell lysates. siSNX4 sufficiently decreased the levels of SNX4, BACE1, APP, and BACE1-mediated APP-processing products compared with siCTL, and the decrease of indicated protein levels was rescued with SNX4. c Quantification of Western blot band intensities. The graphs display the immunoreactivity to BACE1, APP, sAPPβ, and Aβ antibodies, normalized to β-actin (**p < 0.01, ***p < 0.001). d Primary neurons were cotransfected with BACE1-HA and siCTL, siSNX4 mixture, or siSNX4 mixture and SNX4. Immunocytochemistry was performed using an anti-HA antibody (green). Scale bar = 10 μm. (JPG 530 kb) [file 13195_2016_232_MOESM5_ESM.jpg]
